# How to evaluate the potential occurrence of intraguild predation

**DOI:** 10.1007/s10493-017-0142-x

**Published:** 2017-06-01

**Authors:** Morgana Maria Fonseca, Marta Montserrat, Celeste Guzmán, Inmaculada Torres-Campos, Angelo Pallini, Arne Janssen

**Affiliations:** 1 Department of Entomology, Federal University of Viçosa, Viçosa, MG Brazil; 2 Institute for Mediterranean and Subtropical Horticulture “La Mayora” (IHSM-UMA-CSIC), 29750 Algarrobo-Costa, Málaga Spain; 3 Estación Experimental de Zonas Áridas (EEZA-CSIC), Carretera de Sacramento s/n, 04120 Almería, Spain; 4 IBED, University of Amsterdam, Science Park 904, 1098 XH Amsterdam, The Netherlands

**Keywords:** Predator–predator interactions, Experimental design, Population dynamics, Food webs, Evolution

## Abstract

Intraguild predation is the combination of exploitative competition and predation among potential competitors that use similar resources. It has the potential to shape population dynamics and community structure. Although there is much empirical evidence for the occurrence of intraguild predation in natural ecosystems, the study of its effects is mainly limited to short-term microcosm experiments. There is, therefore, certain skepticism about its actual significance in nature. A relevant concern is that there is no consensus regarding criteria to evaluate the possible occurrence of intraguild predation in short-term experiments, and methodological differences may therefore underlie apparent inconsistencies among studies. Our purpose here was to evaluate existing criteria to offer guidance for the design of experiments to determine whether two species may potentially engage in intraguild predation. The criteria are based on the condition that intraguild predators need to experience immediate energetic gains when feeding on the intraguild prey. Thus, a relevant experimental design must quantify predation but also fitness benefits of feeding on the other species, i.e. increases in reproduction, somatic growth, or survival.

## Introduction

Intraguild predation (IGP) consists of the combination of exploitative competition and predation among potential competitors that use similar resources (Polis et al. 1989). It has received considerable attention in the theoretical literature since the influential paper of Polis et al. (1989) pointed out its potential to shape population dynamics and community structure (Diehl and Feissel 2000; Mylius et al. 2001; van de Wolfshaar et al. 2006; Ellner and Becks 2011; Hin et al. 2011). Theoretical models predict that IGP results in more complex population dynamics than either competition or predation alone and can lead to exclusion, coexistence or alternative states (Polis and Holt 1992; Holt and Polis 1997). Despite the ubiquity and importance of IGP, empirical evidence for its effects is mainly limited to microcosm experiments, and there exists, therefore, certain skepticism about its actual occurrence and significance in nature (Kindlmann and Houdková 2006; Gagnon et al. 2011). Nevertheless, there are various examples of its occurrence in a wide variety of communities (Polis et al. 1989; Polis and Holt 1992; Rosenheim et al. 1995; Arim and Marquet 2004), and new methods, using primers specific for the IG prey, are increasingly used to demonstrate the occurrence and importance of this interaction in the field (for example, Yang et al. 2017).

**Fig. 1:**
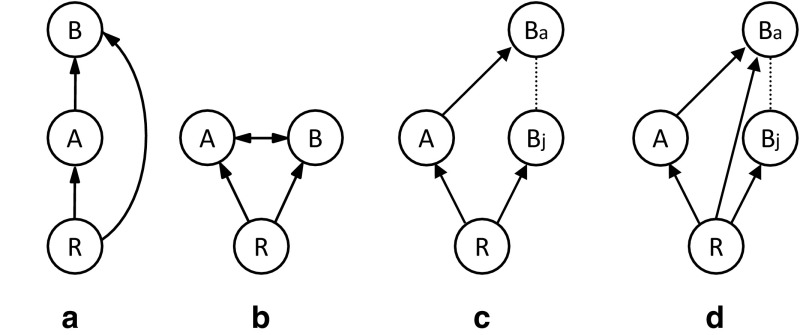
Different types of intraguild predation. The capitals in the circles indicate the shared resource (R), and the two species involved in intraguild predation (*A* and *B*). *Arrows* point from victim to attacker, interrupted lines indicate development. Juveniles and adults are indicated with “j” and “a”, respectively. **a** Simple intraguild predation: species A feeds on the shared resource, species B feeds on the resource and on species *A*. **b** Simple reciprocal intraguild predation: species *A* and *B* feed on the shared resource and on each other. **c** Intraguild predation with complete ontogenetic diet shift: juveniles of species *B* feed on the shared resource, whereas adults feed on species *A*. **d** Intraguild predation with partial diet shift: juveniles of species *B* feed on the shared resource, adults feed on the resource and on species *A*. After Mylius et al. (2001) and van der Hammen et al. (2010)

The simplest system with IGP involves three species: one competitor species (the intraguild predator, IG predator hereafter) that feeds on the other competitor (the intraguild prey, IG prey hereafter) and a shared prey (or shared resource), which is consumed by both competitors (Fig. 1a, Polis and Holt 1992; Holt and Polis 1997). Predictions of the dynamics and coexistence of these three species generally depend on several parameters, such as predation rates, prey preference of the IG predator, ontogenetic diet shifts, and productivity (or carrying capacity of the resource). Whereas there are no publications that systematically investigate the effects of all of these, most publications present predictions as a function of productivity. This is also the parameter that is most easily manipulated experimentally. We therefore focus primarily on this parameter in this paper. Consistent predictions of IGP theory are that coexistence of IG predators and IG prey depends on the productivity level of the resource and the competitive dominance of the IG prey compared to the IG predator (Polis et al. 1989; Diehl and Feissel 2000; Mylius et al. 2001). For coexistence of all three species, the IG prey must be superior at exploitative competition for the shared resource, and even then, coexistence is only possible at intermediate levels of productivity (Polis and Holt 1992, Table 1). If the IG predator is the superior competitor or when productivity levels are high, the IG prey is always excluded (Diehl and Feissel 2000; Mylius et al. 2001, Table 1). At low productivity levels, the best competitor dominates (Holt and Polis 1997; Diehl and Feissel 2001; Mylius et al. 2001, Table 1).

**Table 1:** A comparison of the predicted consequences of competition, intraguild predation (IGP) and reciprocal intraguild predation on species persistence. Shown are the species that will persist at three different productivity levels (Low, Intermediate, High), with A being the superior competitor and B being the inferior competitor

Interaction	Productivity			
	Low^1^	Intermediate	High	Details^2^
Competition	A	A	A	a
IGP with A as IG-predator	A	A	A	b
IGP with B as IG-predator	A	A, B or AB	B	b
Reciprocal IGP	A or None	A or B	A or B	c

These coexistence patterns are mostly based on analytical or numerical stability analyses and invasion criteria (Holt and Polis 1997; Mylius et al. 2001; HilleRisLambers and Dieckmann 2003), but also hold for unstable, paradox-of-enrichment types of dynamics. Holt and Polis state that a Lotka–Volterra model showing unstable dynamics show such high amplitude cycles that populations effectively would go extinct. Possibly, cyclic dynamics increases possibility for coexistence, as was shown for resource competition (Armstrong and McGehee 1980; Huisman and Weissing 1999). ^1^ Low productivity levels allow for the superior competitor to persist with the resource, but not the inferior competitor; ^2^ a: Tilman (1980); b: Holt and Polis (1997), Mylius et al. (2001), Diehl and Feissel (2000), van de Wolfshaar et al. (2006), Hin et al. (2011); c: Priority effects occur, except for intermediately strong trade-offs between feeding on the shared resource or the other predator (HilleRisLambers and Dieckmann 2003). Montserrat et al. (2008, 2012) present experimental evidence for bistability, and show that the inferior competitor can eliminate the superior competitor at low productivity levels, but subsequently also goes extinct

If the two competitors feed on each other, they are engaged in a more complex type of IGP termed reciprocal intraguild predation (Fig. 1b), which is also frequently observed in systems of true predators (Polis et al. 1989; Wissinger 1992; Woodward and Hildrew 2002; Montserrat et al. 2012). When such mutual predation is included in the basic model of IGP, the possibilities for coexistence of the three species are dramatically reduced compared to simple IGP (Table 1, HilleRisLambers and Dieckmann 2003; Montserrat et al. 2012; Schellekens and van Kooten 2012). The predictions are that reciprocal IGP could lead to mutual exclusion or alternative states, in which either one or the other competitor persists alone with the resource, depending on the initial densities (Table 1, HilleRisLambers and Dieckmann 2003; van der Hammen et al. 2010; Montserrat et al. 2012), and there is only limited possibility for coexistence (Schellekens and van Kooten 2012). At low productivity levels, only the best competitor can coexist with the shared resource, whereas priority effects determine which competitor will persist at intermediate to high productivity levels (Table 1, Montserrat et al. 2008; van der Hammen et al. 2010; Montserrat et al. 2012). In the latter case, the competitor that arrives first can build up a population and exclude the other competitor through intraguild predation, thus, the order of invasion is a decisive factor in systems with reciprocal IGP (HilleRisLambers and Dieckmann 2003; Montserrat et al. 2008; van der Hammen et al. 2010; Montserrat et al. 2012).

The theoretical predictions above concern the state of populations in the long-term, when the populations reach stable equilibria or persistent fluctuations. However, laboratory experiments on IGP are frequently only short-term because of experimental logistics (Briggs and Borer 2005; Janssen et al. 2006). Such short-term experiments can only provide qualitative information about whether or not two species are potentially able to engage in IGP (Guzmán et al. 2016). Nevertheless, long-term dynamical predictions are commonly inferred from these short-term experiments and consequently may lead to inaccurate interpretations (Brown et al. 2001; Hastings 2004; Briggs and Borer 2005). Recently, Guzmán et al. (2016) introduced another relevant concern about the methods used in these short-term experiments, that is, there is no consensus regarding criteria to evaluate the occurrence of IGP, and thus, methodological differences may underlie apparent inconsistencies among studies. These authors proposed criteria to evaluate IGP in predatory mites, but these criteria are easily generalized to other arthropod systems, e.g., aquatic arthropods (Wissinger and McGrady 1993; Crumrine 2005), arthropod pests (Rosenheim et al. 1995) and spiders (Polis and McCormick 1986). However, we think that the criteria deserve some further clarification given the number of publications on this topic, both within acarology and in ecology in general. Our purpose here is therefore to further explore such criteria in order to offer guidance on how to design appropriate experiments to determine whether or not two species may potentially engage in IGP.

## IGP or interspecific killing

The experimental design proposed by Guzmán et al. (2016) to evaluate the occurrence of IGP consists of all combinations of two main factors resulting in four treatments: the presence or absence of the IG predator, the presence or absence of the shared resource, with the IG prey always present. Such a design would allow assessing the mortality rate of IG prey and it assumes previous knowledge that both IG prey and IG predator feed on the shared resource. However, this design does not allow assessing one further prerequisite for the occurrence of IGP, which is that the IG predator does not only kill the IG prey, but also feeds on it. This may seem obvious, but predators may simply kill other predators without feeding on them (Palomares and Caro 1999), in which case the interaction is qualified as interspecific killing (an extreme form of interference competition). In many systems, especially those with large predators, it is possible to distinguish interspecific killing from intraguild predation through observing the IG predator consuming the IG prey (e.g. Björklund et al. 2016). We can safely assume that this consumption results in nutritional or energetic gains in natural systems; if not, IG predators would be selected not to feed. However, in other systems, including those with predatory mites and other small arthropods, observing consumption of prey is often difficult because of the size of the study organisms and the low frequency of predation events. Therefore, prey and predators are often confined and the mortality of the prey is assessed after some period through quantification of their shriveled remains, but it is then unclear whether the predator only killed the prey or also fed on it. It is with such a common experimental set-up in mind, that we suggest the experiments below.

Thus, predation of the IG prey by the IG predator should provide direct nutritional and energetic gains that result in increased growth, reproduction or survival of the IG predator (Polis et al. 1989). Although this is essential, many publications, including some of our own, do not present it as an important component of IGP. It is relatively easy to assess gains when IG prey are an equally good or better food source than the shared resource. For instance, the larval performance of the ladybird *Harmonia axyridis* was equal on intraguild prey and the shared resource (Yasuda and Ohnuma 1999; Cottrell 2004; Sato et al. 2008), and two predatory mites performed better when feeding on each other’s immature stages than on the shared resource (Buitenhuis et al. 2010). However, there is one potential complication in assessing gains when the IG prey is a trivial component in the diet of the IG predator, and removing it from the IG predator’s diet may not result in any direct effects on the latter’s fitness. However, consuming the IG prey should result in direct positive fitness effects in the absence of other food, and this absence will occur as a result of competition for the shared resource between the IG prey and IG predator. Even when it is a minor diet component when other prey are present, IG prey may contribute to the persistence of IG predators in periods of food scarcity (Janssen et al. 2006).

## Experimental assessment of the occurrence of intraguild predation

Guzmán et al. (2016) suggest as main criterion that short-term predation experiments should show that IG predators attack both IG prey and shared resource when both are present. If they do not attack the IG prey or do not feed on the shared resource, the authors conclude that the two species are not engaged in IGP, but are involved in competition for resources or a predator–prey interaction, respectively. With hindsight, we think that this criterion is somewhat too strict. It is certainly true that the two predators are involved in intraguild predation when the IG predators attack both the IG prey and the shared resource in such experiments. However, IGP can still occur in nature when IG predators do not feed on both the IG prey and the shared resource within the arbitrary duration of an experiment. Actually, the strength of predation and competition will vary over time, depending on the densities of the shared resource and the IG prey. Accordingly, if IG predators forage only on the shared resource in the presence of the IG prey, this does not mean that there are no circumstances in which they will feed on the IG prey.

For example, Holt and Polis (1997) argued that if IG predators follow the rules of optimal diet theory, the IG prey would be dropped from the IG predator’s diet when the resource is abundant and a better diet than IG prey, allowing for coexistence to occur. After being dropped from the diet, IG prey populations would recover, resource levels would decrease as a result of the combined comsumption by the IG prey and IG predator, and the IG prey would again be included in the IG predator’s diet (Krivan 2000). In this way, adaptive foraging of the IG predators would result in temporal changes in the diet of the IG predators, with alternating periods in which the IG predator feeds on the IG prey and the shared resource and periods in which the IG predators would only feed on the resource. Similar diet restrictions and expansions are expected when the IG prey is a better diet, with the shared resource being excluded or included from the diet depending on the densities of the IG prey (Krivan and Diehl 2005). Although the system of IG predators, IG prey and shared resource will then go through periods in which there is effectively no IGP, the long-term dynamics of the system are still determined by the occurrence of IGP (Krivan and Diehl 2005).

A second example of IGP where consumption of resource and IG prey by the same individual is separated in time is found in systems with ontogenetic diet shifts (Polis et al. 1989; Mylius et al. 2001; van de Wolfshaar et al. 2006; Hin et al. 2011). Organisms are also not necessarily restricted to one ecological role during their entire lives (Polis et al. 1989; Mylius et al. 2001). Hence, IG predators may sometimes feed on the IG prey, and feed on the common resource at other times. In its most extreme form, adult IG predators may feed on IG prey, whereas juvenile IG predators consume resource only (Fig. 1c). Although each ontogenetic stage separately is involved in one interaction only (adult IG predators are predators of the IG prey, juveniles are competitors), each individual is involved in both interactions, but not during the same life stage or age. Nevertheless, this interaction also falls under the definition of IGP (Polis et al. 1989).

Besides these two examples, there are also conditions in which the consumption of IG prey and shared resource by the IG predator within an experiment remains undetected. Consider no diet preference and a satiating functional response in the IG predator. When the density of the shared resource is high relative to that of the IG prey, attacks on the IG prey will be low, simply because the IG predators spend time handling the shared resource or because they become satiated when feeding on the abundant shared resource. As a result, the observed mortality rate of the IG prey may be low and not significantly different from its natural mortality in the absence of IG predators. Hence, a non-significant difference in the observed mortality rates of the IG prey in the presence or absence of the IG predator does not necessarily prove that IGP does not occur at lower densities of the shared resource or higher densities of the IG prey. This could perhaps be solved by increasing the number of replicates of the experiment, which might ultimately result in a significant difference in mortality of the IG prey in the presence vs the absence of the IG predator. However, there are better alternatives, for example, changing the densities of IG prey or the shared resource. The most extreme case of reducing densities is setting them to zero, which results in exactly one of the treatments suggested below: the IG predator with the IG prey in the absence of the shared resource.

## Criteria for assessing the possible occurrence of intraguild predation

**Fig. 2:**
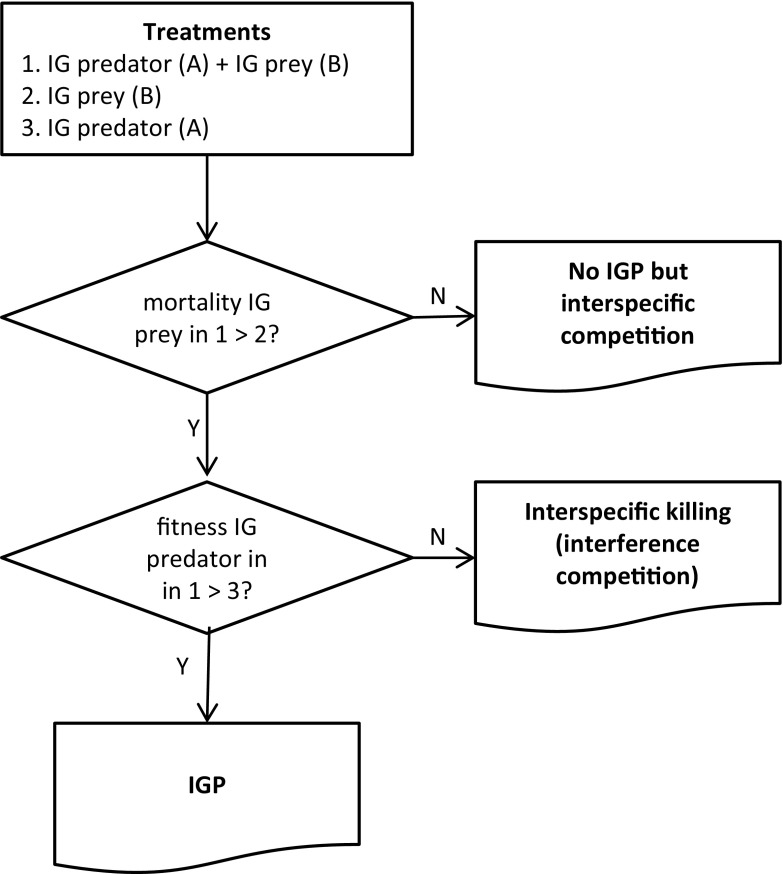
A flow chart of the experiments needed to assess the potential occurrence of intraguild predation in systems where direct observations of IG predators consuming IG prey are difficult to obtain. *Letters A* and *B* refer to the letters used in the text, with *A* being the stage of the species that is the suspected IG predator, and *B* being the stage of the species that is the suspected IG prey. It is assumed that some stage of both the IG prey and IG predator feed on the shared resource, but not necessarily the same stage as tested here. If this is not known, extra predation experiments with the shared resource and the IG prey or IG predator need to be done

Based on the above arguments, we suggest that, to ascertain the potential occurrence of IGP between two competitors A and B, a relevant experimental design must quantify predation but also consumption, or otherwise increased reproduction, somatic growth or survival of one species feeding on the other (i.e. immediate fitness benefits). We assume that it is known that both species attack the shared resource and can grow, develop and reproduce on it (i.e. they are competitors). If not, extra experiments need to be done to ascertain this. We thus suggest the following design with three treatments (Fig. 2), with A being the possible IG predator and B being the possible IG prey, each of the appropriate stage: (1) species A with species B; (2) the same stage of species B alone; (3) the same stage of species A alone. When the mortality of B in the first treatment is significantly higher than in the second treatment, it can be concluded that A kills B. This interaction can then be intraguild predation or interspecific killing (Fig. 2). If the mortality of species B is not higher in the first treatment, the species are involved in resource competition. Subsequently, if the fitness of A in the first treatment is higher than in the third treatment, it is concluded that A consumes B and benefits from it: A is the intraguild predator and B is the intraguild prey (Fig. 2). If the fitness in the first treatment is not higher than in the third treatment, the interaction can be classified as interspecific killing or interference competition.

To verify whether killing or predation occurs may often take less time than to demonstrate effects of feeding on the IG prey on reproduction, growth and survival of the IG predator. In predatory mites, for example, predation of the IG prey can be assessed within one day, but it may take several days to observe effects on IG predator oviposition, and even longer to perceive effects on survival and development. The experiments proposed here may therefore take more time than the simple predation experiments that are often done.

When reciprocal intraguild predation is suspected (Montserrat et al. 2012), these experiments should be repeated with the suspected stage of species B serving as predator and the suspected stage of species A as prey. In cases of ontogenetic diet shifts (Fig. 1c, d), the appropriate stage of the IG predator (for example the adults) should be tested, which is not the stage that also feeds on the shared resource (Fig. 1c).

## Re-evaluation of the reviewed literature

Guzmán et al. (2016) evaluated 18 papers on IGP between phytoseiids, and found that three of them met their criteria. Seven of these 18 papers met the criteria suggested here: they reported the predation of the IG predator on the IG prey as well as some fitness benefit due to this predation (Meszaros et al. 2007; Momen and Abdel-Khalek 2009; Momen 2010; Buitenhuis et al. 2010; Mendel and Schausberger 2011; Montserrat et al. 2012; Gotoh et al. 2014). The other papers, including some of our own (Cakmak et al. 2006; Montserrat et al. 2008), did not include the treatment with the potential IG predator alone to establish fitness benefits (i.e. the comparison of the first and the third treatment as suggested above). The experiments of Guzmán et al. (2016) do involve all necessary treatments, but there is no specific comparison of the oviposition rate of the IG predator in the presence or absence of the IG prey (as measure for fitness benefit). However, the reported significant effect of the interaction between the presence/absence of shared resource and IG prey on IG predator oviposition suggests that there is such a fitness benefit.

## The evolution of intraguild predation

As explained above, besides the indirect benefit of decreasing the population of potential competitors, thus freeing resources, IGP also results in direct energetic benefits (Polis et al. 1989). If the killing of competitors is not profitable and does not provide any energetic gains to the aggressor, they are not engaged in intraguild predation but in an extreme form of interference competition. Interference is a common interaction among competitors and a frequent coevolutionary response to exploitation competition (Simberloff 1982; Connell 1983; Schoener 1983; Polis 1988). Eliminating the ability of a competitor to exploit resources through interference is one possible evolutionary response to lessen potential harmful effects of exploitation competition (Polis 1988). Intraguild predation may be the next step in the evolution of interference between predators (Polis et al. 1989). There will probably be selection for those predators that do not only kill the competitors, but also feed on them, even if this only results in small increases in fitness. Subsequently, selection can act on the IG predators to be more effective at converting IG prey. Thus, the immediate energetic gains distinguish IGP from traditional concepts of competition and its multiple fitness benefits further facilitate the evolution of this interaction (Polis 1988; Polis et al. 1989). Another advantage of being able to feed on the IG prey is that it may increase the persistence of IG predators in periods of scarcity of the shared resource (Janssen et al. 2006).

An alternative evolutionary route to IGP occurs in a tritrophic system with a predator, a prey and the food of the prey. When a predator subsequently evolves to include the food of the prey into its diet, the predator and the prey become involved in intraguild predation. Although it is difficult to know which of the two routes have historically been followed, there are perhaps some indications that this has happened, for example when the IG prey is a better food source for the IG predator than the shared resource is. For instance, in a system with reciprocal IGP between predatory mites used to control the western flower thrips, *Amblyseius swirskii* had higher oviposition rates and a faster development time when feeding on immature stages of the IG prey than on the shared resource (Buitenhuis et al. 2010). Furthermore, a mixed diet of IG prey with the resource may also be important to fulfill energy requirements or supplement nutritional deficiencies, for example, of protein for IG predators that feed primarily on plants which mostly consist of carbohydrates (Polis et al. 1989).

## The importance of intraguild predation for dynamics and persistence

The criteria suggested above will reveal whether IGP can occur between species pairs, but the next question is whether this IGP is important for population dynamics and coexistence of the species involved. This can only be evaluated in replicated, long-term population experiments and invasion experiments, of which there are not many around (Morin 1999; Diehl and Feissel 2000; Montserrat et al. 2008, 2012; Hiltunen et al. 2013; Wilken et al. 2014). These experiments serve to discriminate between IGP, classical competition and predation (Table 1). With resource competition, the species that reduces the level of resources the most wins (Table 1, Tilman 1980; but see Armstrong and McGehee 1980; Huisman and Weissing 1999). In classical tritrophic predator–prey systems, predators only persist with the resource and the prey when the productivity of the system is sufficiently high (Oksanen et al. 1981). As outlined above, in systems with IGP, coexistence depends on both productivity levels and on which of the competitors is the IG predator (Holt and Polis 1997; Diehl and Feissel 2000; Mylius et al. 2001). It furthermore depends on ontogenetic diet shifts; if the intraguild predator can only survive or reproduce when feeding on the IG prey (Fig. 1c), they can only persist in the presence of IG prey and at sufficiently high productivity levels, similar to a classical predator–prey system (Mylius et al. 2001; Hin et al. 2011). More complex forms of diet shifts, where adult and juvenile IG predators differ gradually in the rate of attack of the resource (Fig. 1d), can give rise to various alternative equilibria, depending on the importance of predation relative to competition (Hin et al. 2011).

Theory predicts that the possibilities for coexistence of species involved in IGP are limited and depend on productivity levels, on which species is the superior competitor, and on the occurrence of reciprocal IGP. In contrast, IG prey and IG predators co-occur frequently enough in nature to allow the observation that IGP is a common interaction (Arim and Marquet 2004). Various causes have been suggested for this discrepancy between theory and reality, such as habitat heterogeneity or the presence of refugia for IG prey (Finke and Denno 2002; Janssen et al. 2007; Ferreira et al. 2011; Lemos et al. 2015), and flexible foraging behaviour of the IG-predator (Kondoh 2003). Furthermore, the various interactions occurring in complex food webs may decrease the strength of the interaction, resulting in increased coexistence of IG prey and IG predators (Fagan 1997). The importance and consequences of IGP for species coexistence ultimately need to be assessed in population-dynamical experiments or invasion experiments at various productivity levels (e.g. Morin 1999; Diehl and Feissel 2000, 2001; Montserrat et al. 2008; Wilken et al. 2014). Moreover, the effects of age or stage structure on the success of invasion of one species into a population of the other species should be studied experimentally (Montserrat et al. 2012).

Predator–prey theory has historically focused on interactions between individual prey and predator species and largely ignored the possible effects of predator-predator interactions (Holling 1965; Connell 1983; Kareiva 1994; Losey and Denno 1998). It has become clear in the last 3 decennia, however, that it is paramount to understand how a complex of predators can interact to affect each other’s behaviour and dynamics, as well as their subsequent effects on behaviour and population dynamics of the shared prey (Polis et al. 1989; Lima 1992; Kareiva 1994), including the consequences of this for biological pest control (Rosenheim et al. 1995; Janssen et al. 2006). Although it is important to identify the occurrence of IGP, we suggest that further research should consider the magnitude (interaction strength), the population dynamics and the coexistence of the interacting species, which may differ profoundly from that of systems of competing species or of predators and prey. The challenge is to investigate the effects of IGP in natural systems more rigorously, and laboratory tests of the possible occurrence of IGP, as proposed here and elsewhere (Guzmán et al. 2016), are a necessary first step.
